# Interest and effort in learning and performance

**DOI:** 10.1111/bjep.70040

**Published:** 2025-10-06

**Authors:** Laura Kehle, Detlef Urhahne

**Affiliations:** ^1^ University of Passau Passau Germany

**Keywords:** effort, interest, learning outcomes, motivation to learn

## Abstract

**Background:**

Interest and effort are key motivational constructs in educational psychology, yet their interplay in learning remains underexplored. Building on Dewey's (*Interest and effort in education*, 1913) view that effort complements interest in fostering academic achievement, this research examines their relationship across different learning phases in accordance with a process model developed by Thoman et al. (*The science of interest*, 2017).

**Aims:**

The aim of both studies is to empirically examine the interplay between interest and effort as predictors of learning outcomes.

**Sample:**

The sample of Study 1 included 152 university students, and the sample of Study 2 included 120 university students.

**Methods:**

Study 1 comprised two different learning tasks to investigate the relationship between interest and effort. Study 2 made use of a computer‐supported learning environment where additional influences of prior knowledge could be controlled. Interest and effort were assessed before, during, and after the learning tasks.

**Results:**

The results of both studies indicate that interest and effort serve as positive predictors of learning success and actively complement each other throughout the learning process.

**Conclusion:**

Findings suggest that although interest stimulates initial engagement, effort is essential for sustained learning, supporting Dewey's view of their interdependence. Educators should foster both interest and effort to enhance learning outcomes.

## INTRODUCTION

The concept of interest has developed into a key explanation for motivated behaviour in educational psychology (Renninger et al., 2019; Renninger & Hidi, 2022; Urhahne & Wijnia, 2023). This is supported by research examining interest as a predictor of academic achievement (Jansen et al., 2016; Schiefele et al., 1992), test scores (Jansen et al., 2016), and task mastery (Rotgans & Schmidt, 2017). Additionally, fostering enduring interests is considered a key objective of school‐based education (Harackiewicz & Hulleman, 2010). Research on interest often focuses on discovering connections to characteristics of the learning context, such as instructional type (Lee & Boo, 2022), and its relationship with other motivational constructs, like self‐efficacy (Schweder & Raufelder, 2022).

The theoretical understanding of interest has been strongly influenced by philosopher and educationalist John Dewey, who is considered the pioneer of modern interest theory (Schiefele, 1991). Dewey (1913) placed particular emphasis on the relationship between interest and effort in education. Although these two constructs differ greatly in their affective connotations, effort in thinking was not regarded as the opposite of interest. Rather, mental effort was an integral part of a growth process that fosters deeper interest.

However, according to contemporary theoretical perspectives, interest and effort still appear, to some extent, to be opposed. Interest is considered an intrinsically motivated state, associated with positive emotions (Schiefele, 1991) and an effortless focus of attention (McDaniel et al., 2000). In contrast, effort represents the intensity of a motivated state (Brehm & Self, 1989) and is often linked to feelings of unpleasantness when sustained attention is required over extended periods (David et al., 2024).

This research, therefore, examines the empirical relationship between interest and effort in learning in greater depth. The focus is on a detailed analysis of the mechanisms at work and the interplay between these two variables in the learning process, both conceptualized and measured as subjective constructs. Effort is considered a complementary factor that extends the current theory of interest (Renninger & Hidi, 2022; Thoman et al., 2017), suggesting that both motivational constructs can jointly influence learning and academic achievement (Dewey, 1913).

## THEORETICAL BACKGROUND

### Interest

Interest, a key motivational construct in learning, is represented by two different types: individual and situational interest. Individual interest refers to a motivational disposition or orientation that leads a person to engage voluntarily and repeatedly with a particular object over an extended period of time. In contrast, situational interest describes a positively experienced state that arises when a person interacts with a specific object (Hidi & Renninger, 2006; Krapp, 1992; Renninger & Hidi, 2022; Schiefele, 1999; Silvia, 2001). These types of interest operate at different levels: Individual interest reflects habitual or dispositional aspects of personality, while situational interest pertains to immediate engagement with an object. Individual interest often arises from situational interest. This happens through repeated engagement with, or exposure to, a particular object or activity (Hidi & Renninger, 2006; Silvia, 2001). Empirical research indicates that both individual interest (Jansen et al., 2016; Schiefele et al., 1992) and situational interest (Linnenbrink‐Garcia et al., 2013) are positive predictors of academic achievement. In the following, we focus on situational interest, as it reflects learners' immediate engagement with the learning material.

### Interest in the learning process

Situational interest fluctuates as individuals encounter different learning situations. The dynamic nature of interest arises from a person's continuous interaction with their environment. While interest can be actively induced or influenced (Bergin, 1999), it often changes unintentionally due to factors like prior knowledge, personal traits, goal orientations, learning context, and behaviour. Situational interest evolves as these determinants change throughout the learning process (Thoman et al., 2017). As a short‐term state, it can decline very rapidly, which is why it may predict achievement particularly at the beginning of a learning process but ceases to be predictive thereafter (Hidi, 2001; Nuutila et al., 2021; Ochsen et al., 2023).

Within the learning process, three distinct states of situational interest can be identified (Thoman et al., 2017): (1) Triggered interest – occurs before engagement with the learning object. It is a form of situational interest influenced by both the incentives of the learning object and the individual's existing interest. (2) Actual interest – arises during active engagement as a short‐term state, highly dependent on the specific situation, and lasting several minutes while working on tasks. Due to its transient nature, it is often measured at short intervals during task engagement (Hidi, 2001; Ochsen et al., 2023). (3) Experienced interest – represents a retrospective assessment of the learning process, in which individuals reflect on their experiences and evaluate their level of interest. It is shaped by learning outcomes and, in turn, influences individual interest (Thoman et al., 2017). Experienced interest that arises following active engagement has been shown to have a positive effect on long‐term retention (Fastrich et al., 2018).

### Effort

Effort can be understood as the mobilization of resources during learning (Gendolla & Wright, 2009) and encompasses multiple facets like attention, concentration, persistence, and endurance (Trautwein et al., 2006, 2015). Effort is applied in a situation‐specific manner to achieve desired goals (Gendolla & Wright, 2009). Consequently, effort can vary greatly between different learning tasks as well as within a single task (Heemskerk & Malmberg, 2020; Trautwein et al., 2015).

Learning with effort can have a highly effective impact on learning outcomes (Jin, 2024; Silm et al., 2020). However, learners often interpret effort as a sign of ineffective learning or lack of ability (Baars et al., 2020; David et al., 2024; Kirk‐Johnson et al., 2019). This misunderstanding arises due to learners' limited insight into their objective performance.

Effort is sometimes described as aversive, despite its potential benefits, yet theoretical and empirical evidence suggests that people primarily avoid unnecessary or wasted effort rather than effort itself. Therefore, effort is only exerted when necessary and justified (Brehm & Self, 1989; Gendolla, 2025; Gendolla et al., 2012; Inzlicht et al., 2018; Richter et al., 2016). However, due to a personal disposition (Mlynski et al., 2025), learned industriousness (Eisenberger, 1992), or reward transfer learning (Clay et al., 2022; Kraus et al., 2025), some individuals are more inclined to invest effort willingly – even when it is not strictly necessary or justified. Dewey (1913, p. 59) emphasized that a person ‘who associates difficulties and effort with increased depth and scope of thinking will never go far wrong.’ Empirical findings indicate that while more effective learning strategies are often perceived as more demanding and less conducive to learning (Kirk‐Johnson et al., 2019), effort remains a stable and positive predictor of academic achievement (Jin, 2024; Silm et al., 2020).

Effort, like interest, can be classified into distinct subjective phases within the learning process: (1) Planned effort – the subjective willingness to exert effort before engaging with a learning object, based on prior experience and beliefs. At this stage, the precise effort required cannot yet be determined. Instead, planned effort provides an estimate of the resources needed to complete the task and whether the investment of these resources is justified. According to motivational intensity theory (Brehm & Self, 1989), the assessment of justified effort represents the motivation invested in a task. (2) Experienced actual effort – hereafter referred to as actual effort – the subjective perception of resource expenditure while working on the learning task. Since it is experienced in real time as resources are utilized, it can be assessed more accurately. Here, effort serves as a means of evaluating whether the resources expended are proportionate to the desired goal and whether the activity should be continued or abandoned. As task difficulty increases, more effort is invested, provided the expenditure of resources remains justified (Brehm & Self, 1989). (3) Experienced effort – a retrospective, summative evaluation of the learning process. This subjective perception of effort is influenced by learning outcomes and by attributions of the outcomes to one's own abilities. Effort at this stage is a double‐edged sword (Covington & Omelich, 1979); while strong learning effort is generally desirable, it can also be interpreted as an indication of low ability. It should be noted that these phases refer to subjective effort, that is, learners' subjective perception of their effort, and do not capture objectively measurable effort. Subjective and objective effort rely on different processes and can be dissociated (Bermúdez & Massin, 2022; Bijleveld, 2018; Décombe et al., 2022; Halperin & Vigotsky, 2024; Putz et al., 2024). Due to the various influencing factors across different phases of the learning process, assessments of subjective effort can vary considerably.

### Effort and interest in the learning process

Dewey (1913) argued that effort complements interest in the learning process and should be regarded as part of a shared growth activity. However, only a few studies have examined interest and effort together, and in those that have, the focus has not been on the mutual relationship between the two constructs. Instead, interest has often been treated as a control variable (Fütterer et al., 2022) or analysed as a component within a broader theoretical model (Akhtar & Firdiyanti, 2023).

There are, however, theoretical models that address interest and effort in a combined framework. For example, the CONIC model (i.e., the Conscientiousness × Interest Compensation model) has guided and inspired in‐depth investigations into the extent to which individual interest predicts academic effort (Trautwein et al., 2025). Moreover, the concept of grit, consisting of perseverance of effort and consistency of interest, has enabled the joint investigation of interest and effort on a personality level (Duckworth et al., 2007; Duckworth & Quinn, 2009). Research has shown that grit is moderately associated with performance, and that the perseverance of effort facet tends to be a stronger predictor of academic performance than consistency of interest (Credé et al., 2017). However, grit primarily reflects stable, person‐level characteristics and is typically studied in relation to long‐term outcomes. An in‐depth investigation of interest and effort as distinct, dynamic variables within the learning process, as well as their interplay, remains to be addressed.

**FIGURE 1:**
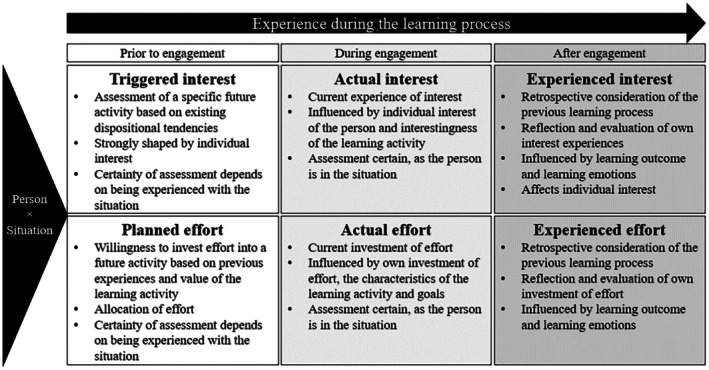
Different phases of interest and effort over the course of the learning process inspired by Thoman et al. (2017).

Building on Thoman et al.'s (2017) process model, this study seeks to explore the phases of interest and effort during the learning process in greater detail (see Figure 1). It is assumed that the interplay between interest and effort is shaped by an interaction between situational and personal factors, aligning with fundamental principles of motivation psychology (Heckhausen & Heckhausen, 2018; for an overview, see Urhahne & Wijnia, 2023). This interaction influences the assessment of triggered interest and planned effort, actual interest and actual effort during learning, and the subsequent evaluation of the learning experience.

Interest can and should facilitate the exertion of effort in learning (Jansen et al., 2016; Marsh et al., 2005). The primary and fundamental function of interest is to foster exploration, which consequently enhances learning (Izard, 2007; Silvia, 2006). Through its value‐related valences (Schiefele, 1991), interest enhances the perceived importance of the learning activity or its outcome. A higher subjective value motivates individuals to invest greater effort in the learning activity to achieve their desired learning goals. Additionally, through its feeling‐related valences (Schiefele, 1991), interest fosters a positive mood (Gendolla, 2000). Individuals in a positive mood tend to perceive the required effort as lower and feel more capable of exerting the necessary effort to reach their goals compared to those in a negative mood (Gendolla, 2025). Thus, a higher level of interest leads to prolonged mobilization and investment of effort, reduces the aversive aspects of effort, and strengthens effort as a predictor of academic achievement throughout the learning process.

Triggered interest is particularly strong at the beginning of the learning process and predicts achievement at this early stage, activated by incentives of the learning object and a person's existing interests (Thoman et al., 2017). At this point, effort is not yet consciously perceived, as it refers to the estimation and justification of the required resources, which have not been mobilized or applied (Brehm & Self, 1989). Since situational interest is a short‐term state, it declines without further triggering, and consequently, should no longer predict achievement at later stages of the learning process (Hidi, 2001; Nuutila et al., 2021; Ochsen et al., 2023). In contrast, effort becomes more salient as the learning process progresses. According to motivational intensity theory (Brehm & Self, 1989), effort reflects the actual mobilization and application of resources. These resources, such as attention and concentration, are invested only once learners engage more deeply with the task. As a result, effort becomes increasingly visible at later stages of learning.

## THE PRESENT STUDIES

This article reports on two studies that examine interest and effort as predictors of learning outcomes across different learning phases. The first study investigates the interplay between interest and effort using two short‐term memory tasks, which require increased effort for successful completion. The second study analyses the relationship between interest and effort within a computer‐based learning environment, while controlling for prior knowledge to examine any potential confounding effects (Simonsmeier et al., 2021).

Effort is often surveyed only as experienced effort after task completion (Järvinen et al., 2022; Symonds et al., 2021), or it is unclear at which stage of the learning process effort is assessed. In our studies, interest and effort are measured both prospectively and in real‐time during the learning process. Identical instruments are used to subjectively assess the variables ‘triggered interest’, ‘actual interest’, ‘planned effort’, and ‘actual effort’ in tasks related to knowledge application and knowledge acquisition (Study 1) and in self‐regulated learning within an interactive computer‐based environment (Study 2). Standardized tests (Study 1) and multiple‐choice tasks (Study 2) serve as objective measures of test achievement. We refrained from including experienced interest and experienced effort in the explanation of test achievement to avoid possible bias from participants' success or failure in the task.

### Hypotheses

The aim of both studies is to empirically examine the interplay between interest and effort as predictors of learning outcomes, as hypothesised by Dewey (1913). Based on the theoretical framework and the extension of the process model by Thoman et al. (2017), the following hypotheses are formulated for both studies:

Hypothesis 1 Interest is a positive predictor of learning success. A high level of triggered or actual interest is expected to enhance learning outcomes.

Hypothesis 2 Effort is a positive predictor of learning success. A high level of planned or actual effort is expected to enhance learning outcomes.

Hypothesis 3 Triggered interest is a stronger predictor of learning success than planned effort. At the beginning of the learning process, interest is expected to play a more significant role in learning than planned but unrealized effort.

Hypothesis 4 Actual effort is a stronger predictor of learning outcomes than actual interest. During the learning process, effort is expected to be more critical to learning success than interest.

In addition to these hypothesized direct effects, we also tested potential indirect effects. Since these are determined by the underlying direct paths and are less well established in theory, we did not formulate explicit hypotheses for them.

## STUDY 1

### Method

#### Sample

A total of *N* = 152 students (69.7% female, age: *M* = 22.24 years, *SD* = 2.47) from a German university took part in the data collection for the first study. An a priori power analysis was conducted with semPOWER (Moshagen & Bader, 2024), which resulted in a minimum sample size of 89 participants assuming typical conditions for conducting structural equation modelling (Cronbach's *α* = .05, 1 − *β* = .80, RMSEA = .04, *df* = 707). The students were on average in the *M* = 5.95 (*SD* = 3.61) semester and were enrolled in various degree programmes. They provided informed consent and received compensation of 10 euros for the 1‐hour examination.

#### Materials

Triggered interest was assessed with six items (e.g., ‘I look forward to working on such tasks’; based on the definition by Hidi & Renninger, 2006) after the respective learning task was explained and a sample task was shown. The reliability of the 5‐point Likert scale was satisfactory for both learning tasks (.90 ≤ *α* ≤ .94).

Actual interest was measured during task processing with six items at two different measurement times (e.g., ‘At the moment I am… enthusiastic – indifferent’; based on the definition by Hidi & Renninger, 2006). The items were answered using a 5‐stage semantic differential to save response time and appropriately represent the current experience in the learning process. The semantic differential proved to be reliable for both learning tasks (.84 ≤ *α* ≤ .91).

Experienced interest was measured with six items (e.g., ‘I enjoyed working on these tasks’; based on the definition by Hidi & Renninger, 2006) after the respective learning task had been completed. The reliability of the 5‐point Likert scale was satisfactory for both learning tasks (.94 ≤ *α* ≤ .95).

Planned effort was recorded using six items (e.g., ‘I don't want to give up on the tasks, even if I'm struggling’; based on the definition by Gendolla & Wright, 2009). The respective learning task was explained beforehand and a sample task was shown. The reliability of the 5‐point Likert scale was satisfactory for both learning tasks (.86 ≤ *α* ≤ .87).

Actual effort was measured during task processing using a 5‐level semantic differential (e.g., ‘At the moment I am… attentive – distracted’; based on the definition by Gendolla & Wright, 2009). In each case, six items were used at two different measurement times. The semantic differential was reliable for both learning tasks (.84 ≤ *α* ≤ .89).

Experienced effort was measured using six items (e.g., ‘I did not give up on the tasks, even when I was struggling’; based on the definition by Gendolla & Wright, 2009) after both learning tasks were completed. The reliability of the 5‐point Likert scale was satisfactory for both learning tasks (.87 ≤ *α* ≤ .88).

In addition, confirmatory factor analyses were used to test whether actual interest and actual effort change structurally during the learning process. This was not the case. The model fit of a single‐factor model across both measurement points was comparable to that of a two‐factor model separated by measurement point. A Chi‐square difference test (Kline, 2011) was not significant for either actual interest (CPT: $\chi_{D}^{2}\left(1\right)$ = .121, *ns*; MPT: $\chi_{D}^{2}\left(1\right)$ = .126, *ns*) or actual effort (CPT: $\chi_{D}^{2}\left(1\right)$ = 2.844, *ns*; MPT: $\chi_{D}^{2}\left(1\right)$ = 2.130, *ns*). For this reason, the data from both measurement times were summarized and latent variables for actual interest and actual effort were formed across both measurement times.

Learning success was assessed using a knowledge application task and a knowledge acquisition task. The concentration–performance test (CPT 6–13; Düker & Lienert, 2001) is a knowledge application task in which the participants solve mental arithmetic problems. The CPT consists of nine blocks, each with 20 arithmetic problems. The maximum completion time for each block is 2 min. The ‘Memory for Persons’ test (MPT; Pahlke & Bulla‐Hellwig, 2002) is a knowledge acquisition task in which participants memorize 10 unfamiliar faces with names and occupations within 1 min. The test was modified so that it could be presented in three blocks to allow interim measurements of current experience. After each block, a distractor task (number‐connection test; Oswald, 2016) was used to avoid possible recency effects and to increase the difficulty of the memorization task. Learning success was measured for each learning task as the total number of correct answers. The reliability of the tests was adequate (*α*_CPT_ = .96; *α*_MPT_ = .58).

#### Design and procedure

During the 60‐minute session, the participants completed a self‐description questionnaire in small groups and worked on the two learning tasks. The order of the two tests was systematically varied to avoid sequence effects. At the beginning, the CPT or MPT was explained according to the test instructions. Participants then completed a sample task to ensure that they knew what to expect and how to complete the tasks. Afterwards, triggered interest and planned effort were measured. The participants then worked on the two learning tasks in three blocks of time. In a short break between the blocks, the participants answered the semantic differential for actual interest and actual effort. In the CPT, the time interval between the measurements was about 6 min and in the MPT about 3 min. At the end of the learning tasks, experienced interest and experienced effort were measured.

#### Statistical analysis

The data were analysed using SPSS 24.0 and AMOS 24.0. The dataset did not contain any missing values, so no estimation procedures were applied. The model fit was evaluated, according to Hu and Bentler (1999), using the relative Chi‐square (cut‐off value *χ* ^2^/*df* ≤ 2), the comparative fit index (cut‐off value CFI ≥ .90), the Tucker–Lewis index (cut‐off value TLI ≥ .90), the root mean square error of approximation (cut‐off value RMSEA ≤ .08), and the root mean square residual (cut‐off value SRMR ≤ .08). For the SEM analyses, all constructs were modelled as latent variables and were permitted to covary with each other. To avoid multicollinearity, we did not include experienced motivational states in the models as the variance inflation factor (VIF) value for actual interest and experienced interest for CPT exceeded the recommended threshold (VIF ≤ 2.5; Johnston et al., 2018). To test Hypotheses 1 and 2, linear structural equation modelling was conducted for both learning tasks. To test Hypotheses 3 and 4, a comparison of the *β*‐coefficients was performed using a structural equation model (SEM) re‐parameterization approach. Similar to a Wald test, a likelihood ratio test (LR test) was used to test whether the *β*‐coefficients differ significantly from each other (Kwan & Chan, 2011).

### Results

The descriptive statistics and the Pearson correlations between the variables are presented in Table 1, separated by learning tasks. Interest and effort correlate positively with test achievement for both learning tasks. The relationships between the motivational variables and achievement are more pronounced in the CPT than in the MPT.

**TABLE 1:** Descriptive statistics and correlation coefficients among variables in Study 1.

Variables	*M*	*SD*	1	2	3	4	5	6	7
CPT									
1. Triggered interest	2.92	1.14	—						
2. Actual interest	3.03	.75	.66**	—					
3. Experienced interest	2.71	1.12	.76**	.81**	—				
4. Planned effort	4.35	.63	.37**	.23**	.29**	—			
5. Actual effort	3.79	.78	.27**	.52**	.46**	.31**	—		
6. Experienced effort	3.97	.85	.21**	.41**	.42**	.38**	.68**	—	
7. Test achievement	59.45	28.93	.53**	.45**	.45**	.15*	.31**	.24**	—
MPT									
1. Triggered interest	3.64	.82	—						
2. Actual interest	3.78	.64	.38**	—					
3. Experienced interest	4.04	.81	.58**	.69**	—				
4. Planned effort	4.46	.56	.34**	.31**	.32**	—			
5. Actual effort	4.16	.64	.21**	.55**	.50**	.54**	—		
6. Experienced effort	4.42	.60	.17*	.47**	.52**	.59**	.72**	—	
7. Test achievement	24.86	3.07	.12	.22**	.27**	.12	.36**	.30**	—

*Note*: *N* = 152.

* *p* < .05.

** *p* < .01.

#### Interest and effort as predictors of learning outcomes

To investigate the relationships in more detail, SEMs were computed separately for both learning tasks. The models for both cognitive tasks showed a good fit to the data (CPT: *χ* ^2^/*df* = 1.61; CFI = .92; TLI = .91; RMSEA = .06; SRMR = .07; MPT: *χ* ^2^/*df* = 1.58; CFI = .91; TLI = .89; RMSEA = .06; SRMR = .08).

**FIGURE 2:**
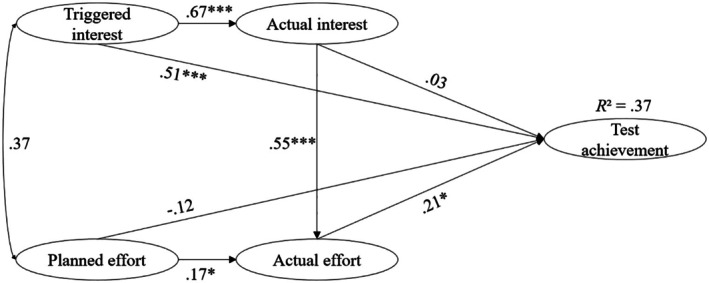
Structural equation model predicting test achievement through interest and effort in Study 1 for CPT. This structural equation model predicts test achievement through interest and effort in the learning process. Statistics are standardized regression coefficients. *p < .05. ***p < .001.

**FIGURE 3:**
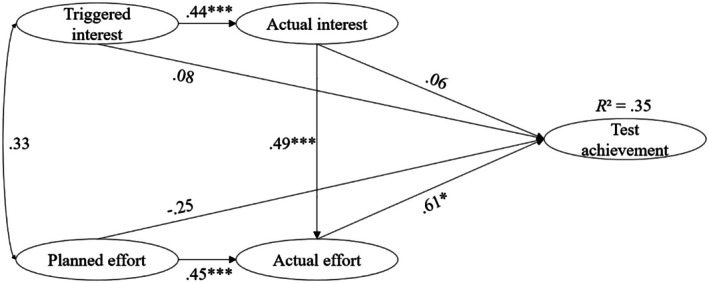
Structural equation model predicting test achievement through interest and effort in Study 1 for MPT. This structural equation model predicts test achievement through interest and effort in the learning process. Statistics are standardized regression coefficients. *p < .05. ***p < .001.

Hypotheses 1 and 2 propose that interest and effort are positive predictors of test achievement. Figures 2 and 3 present the results of the models for the CPT and the MPT. They indicate that for the CPT, both triggered interest and actual effort significantly and positively predict test achievement. For the MPT, only actual effort emerges as a significant positive predictor of test achievement (Table 2).

**TABLE 2:** Comparison of the standardized coefficients with a likelihood ratio test in Study 1.

Parameter	CPT				MPT			
	Unconstrained		Constrained^a^		Unconstrained		Constrained^a^	
	Est.	SE	Est.	SE	Est.	SE	Est.	SE
Triggered interest and planned effort								
*γ*1 (Int. → Ach.)	.536	.057	.261	.047	.090	.086	.088	.049
*γ*2 (Eff. → Ach.)	−.056	.071	.261	.047	.086	.086	.088	.049
*χ* ^2^	.000		22.134		.000		.001	
*df*	0		1		0		1	
Testing H_0_: *γ*_1_ = *γ*_2_								
LR Test	Δ*χ* ^2^ = 22.134, Δ*df* = 1, *p <* .001				Δ*χ* ^2^ = .001, Δ*df* = 1, *p =* .978			
Actual interest and actual effort								
*γ*1 (Int. → Ach.)	.418	.079	.272	.046	.036	.093	.196	.046
*γ*2 (Eff. → Ach.)	.110	.091	.272	.046	.345	.083	.196	.046
*χ* ^2^	.000		3.933		.000		3.599	
*df*	0		1		0		1	
Testing H_0_: *γ*_1_ = *γ*_2_								
LR Test	Δ*χ* ^2^ = 3.933, Δ*df* = 1, *p =* .047				Δ*χ* ^2^ = 3.599, Δ*df* = 1, *p =* .058			

Abbreviations: Eff. → Ach., Effort → Test Achievement; Est., parameter estimate; Int. → Ach., Interest → Test Achievement; *SE*, standard error.

^a^ Constrained model is fitted under H_0_: *γ*_1_ = *γ*_2_.

#### Interest versus effort in predicting learning outcomes

Furthermore, in line with Hypothesis 3, it was examined whether triggered interest is a better predictor of test achievement than planned effort (see Table 2). For the CPT, the LR test of the *β*‐coefficient comparison indicated that the relative effect of triggered interest on test achievement is significantly stronger than that of planned effort. For the MPT, the LR test between initial interest and planned effort revealed no significant differences.

In accordance with Hypothesis 4, it was hypothesized that actual effort would be a better predictor of test achievement than actual interest. When comparing the *β*‐coefficients for the CPT, the LR test indicated that the relative effect of actual effort on test achievement is significantly stronger than that of actual interest. However, for the MPT, no significant differences were found in predicting test achievement Table 2.

An analysis of indirect effects on test achievement was conducted using bootstrapping with 1000 samples. For CPT, the indirect effect of triggered interest through actual interest was estimated as *b* = .657, with a 95% confidence interval of [−.394, 1.609]. The confidence interval includes zero, indicating that this indirect effect is not statistically significant (*p* > .05). The indirect effect of actual interest through actual effort was *b* = 1.606, with a 95% confidence interval of [.121, 3.479]. The indirect effect of planned effort through actual effort was *b* = .480, with a 95% confidence interval of [.030, 1.597]. As both confidence intervals do not include zero, these indirect effects are statistically significant (*p* < .05). For MPT, the indirect effect of triggered interest through actual interest was estimated as *b* = .067, with a 95% confidence interval of [.014, .178]. The indirect effect of actual interest through actual effort was *b* = .185, with a 95% confidence interval of [.030, .471]. The indirect effect of planned effort through actual effort was *b* = .307, with a 95% confidence interval of [.053, .894]. As all confidence intervals exclude zero, all indirect effects are statistically significant (*p* < .05).

## STUDY 2

To gain a better understanding of the interplay between interest and effort in the learning process, a follow‐up study was conducted based on the previous findings. The same hypotheses as in the first study were tested in a computer‐based learning environment. To control for the potential influence of prior experience, the participants' prior knowledge was assessed.

### Method

#### Sample

A total of *N* = 120 students (83.3% female, age *M* = 22.60 years, *SD* = 3.66) from a German university took part in the data collection for the second study. As in Study 1, a minimum sample size of 86 participants was calculated, assuming typical conditions (*α* = .05, 1 − *β* = .80, RMSEA = .04, *df* = 747). On average, the students were enrolled in *M* = 5.85 (*SD* = 3.11) semesters and studied in various degree programmes. They provided informed consent and received an allowance of 10 euros for a 1‐hour examination.

#### Materials

The materials used to measure interest and effort were identical to those used in the first study: Triggered interest (*α* = .89), actual interest (.87 ≤ *α* ≤ .90), experienced interest (*α* = .94), planned effort (*α* = .85), actual effort (.83 ≤ *α* ≤ .86), and experienced effort (*α* = .81) were each measured with six items on five‐point rating scales, demonstrating satisfactory reliability. Because the model fit of a one‐factorial model was comparable to that of a two‐factorial model, the two measures of actual interest ($\chi_{D}^{2}\left(1\right)$ = 3.57, *ns*) and actual effort ($\chi_{D}^{2}\left(1\right)$ = 1.51, *ns*) were again combined (Kline, 2011).

To measure prior knowledge, participants were asked to write down all the technical terms relating to the brain that they could think of within 1 minute. Each correct answer was awarded one point.

The learning environment consisted of an interactive computer programme on the topic of the brain, which was developed on the basis of current textbooks. The programme presented facts about the structure of the brain and the functions of different brain regions in three learning sections. Each section contained two illustrations and informational texts of comparable word count (740–742 words) and difficulty (gSMOG 8.49–8.89, McLaughlin, 1969). The students had 6 minutes for each of the three sections of the learning environment to engage with the content.

A knowledge test consisting of 30 multiple‐choice questions was used to measure learning success, which was designed according to the guidelines of Haladyna and Rodriguez (2013). Ten questions with three possible answers were provided for each section of the learning environment, of which one to three could be correct. The number of correct and incorrect answers was balanced in the test to avoid rewarding answer bias. The maximum achievable score was 90 points, and the internal consistency of the knowledge test was satisfactory (*α* = .60).

#### Design and procedure

The study was conducted in small groups in the university's computer rooms. At the beginning of the session, the students indicated their triggered interest and planned effort for the learning environment. This was followed by the prior knowledge test, after which the participants began to engage with the learning environment. After every 6 minutes, the participants were asked twice to complete the semantic differential for actual interest and actual effort. At the end of the session, the knowledge test was administered without a time limit.

#### Statistical analyses

The data were analysed using the SPSS 24.0 and AMOS 24.0 software. The dataset had no missing values, meaning that no variable imputation was necessary. To test the hypotheses, the same analyses were conducted as in Study 1. Prior knowledge was modelled as a manifest variable.

### Results

The descriptive statistics, as well as the Pearson correlations between the variables, separated by learning tasks, are presented in Table 3. The variables for interest and effort correlate moderately to strongly with each other. The relationships between the motivational variables and test achievement are less pronounced and not always significant.

**TABLE 3:** Descriptive statistics and correlation coefficients among variables in Study 2.

Variables	*M*	*SD*	1	2	3	4	5	6	7
1. Triggered interest	3.72	.69	—						
2. Actual interest	3.82	.66	.43**	—					
3. Experienced interest	3.88	.81	.49**	.58**	—				
4. Planned effort	4.18	.63	.45**	.28**	.33**	—			
5. Actual effort	3.90	.60	.27**	.53**	.39**	.30**	—		
6. Experienced effort	4.06	.68	.32**	.32**	.47**	.36**	.59**	—	
7. Test achievement	70.08	6.10	.04	.12	.19*	.11	.30**	.42**	—

*Note*: *N* = 120.

* *p* < .05.

** *p* < .01.

#### Interest and effort as predictors of learning outcomes

To examine the relationships in more detail, prior knowledge was included as a control variable. The SEM model showed an adequate fit to the data (*χ* ^2^/*df* = 1.45; CFI = .89; TLI = .88; RMSEA = .06; SRMR = .08).

**FIGURE 4:**
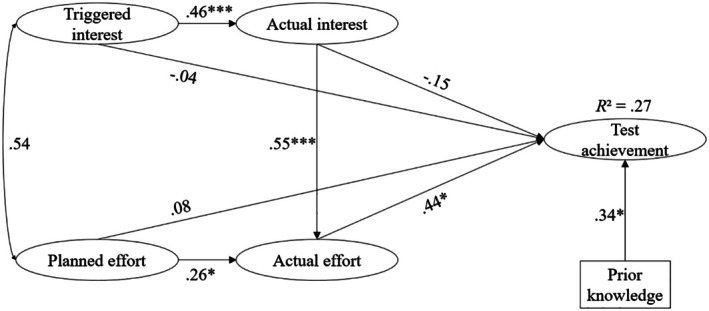
Structural equation model predicting test achievement through interest and effort in Study 2. This structural equation model predicts test achievement through interest and effort in the learning process, controlling for prior knowledge. Statistics are standardized regression coefficients. *p < .05. ***p < .001.

Hypotheses 1 and 2 posit that interest and effort are positive predictors of test achievement. The results in Figure 4 show that only actual effort is a significant and positive predictor of test achievement.

#### Interest versus effort in predicting learning outcomes

In line with Hypothesis 3, it was tested whether triggered interest is a better predictor of test achievement than planned effort (see Table 4). The LR test of the *β*‐coefficient comparison between triggered interest and planned effort revealed that the relative effects on test achievement did not differ significantly.

**TABLE 4:** Comparison of the standardized coefficients with a likelihood ratio test for Study 2.

Parameter	Study 2: Learning environment			
	Unconstrained		Constrained^a^	
	Est.	SE	Est.	SE
Triggered interest and planned effort				
*γ*1 (Int. → Ach.)	−.014	.102	.053	.054
*γ*2 (Eff. → Ach.)	.119	.101	.053	.054
*χ* ^2^	.000		.588	
*df*	0		1	
Testing H_0_: *γ*_1_ = *γ*_2_				
LR Test	Δ*χ* ^2^ = .588, Δ*df* = 1, *p =* .443			
Actual interest and actual effort				
*γ*1 (Int. → Ach.)	−.054	.099	.272	.046
*γ*2 (Eff. → Ach.)	.324	.089	.272	.046
*χ* ^2^	.000		4.523	
*df*	0		1	
Testing H_0_: *γ*_1_ = *γ*_2_				
LR Test	Δ*χ* ^2^ = 4.523, Δ*df* = 1, *p =* .033			

Abbreviations: Eff. → Ach., Effort → Test Achievement; Est., parameter estimate; Int. → Ach., Interest → Test Achievement; *SE*, standard error.

^a^ Constrained model is fitted under H_0_: *γ*_1_ = *γ*_2_.

In line with Hypothesis 4, it was tested whether actual effort is a better predictor of test achievement than actual interest. The LR test revealed that the relative effect of actual effort on test achievement was significantly stronger than that of actual interest on test achievement (see Table 4).

An analysis of indirect effects on test achievement was conducted using bootstrapping with 1000 samples. The indirect effect of triggered interest through actual interest was estimated as *b* = .095, with a 95% confidence interval of [−.195, .452], indicating that this effect is not statistically significant (*p* > .05). The indirect effect of actual interest through actual effort was *b* = .570, with a 95% confidence interval of [.083, 1.684]. The indirect effect of planned effort through actual effort was *b* = .337, with a 95% confidence interval of [.047, 1.218]. As both confidence intervals do not include zero, these indirect effects are statistically significant (*p* < .05).

## DISCUSSION

Interest is considered a key explanatory framework for motivated behaviour within the field of educational psychology (Jansen et al., 2016; Renninger & Hidi, 2022). The ideas of the educational reformer John Dewey shaped the development of interest theory and emphasized its central role in learning. In particular, Dewey (1913) focused on the interplay between interest and effort as components of a unified growth activity to develop sustained interest and successful learning. Interest and effort were not seen as opposites but rather as complementary elements of a reciprocal process. However, this theoretical perspective has faded from academic discourse over the decades. Therefore, this empirical study aims to revive and build upon it.

Initially, two laboratory studies were conducted to investigate the relationship between interest and effort using two different learning tasks. This was followed by a study in a computer‐based learning environment, where additional influences of prior knowledge were controlled. The results indicate that interest and effort serve as positive predictors of learning success and that these two motivational constructs appear to complement each other throughout the learning process.

### Interest and effort as predictors

Interest is a relatively transient motivational state and plays a crucial role in the early phases of learning. According to Hypothesis 1, triggered interest was expected to positively predict test achievement. This association, however, was only evident for CPT. Furthermore, Hypothesis 3 posited that the influence of triggered interest on learning success would be significantly stronger than that of planned effort, a finding that was confirmed for CPT in the results.

Triggered interest is a particularly strong predictor of learning success when learners already possess a stable individual interest in the topic. For domains where learners had the most prior experience (such as mental arithmetic like in CPT), they were better able to assess the potential stimuli of the upcoming task. As a result, triggered interest emerged as a stable predictor of test achievement. However, when interest was less clearly defined before completing the learning task, because learners could not accurately anticipate what to expect (e.g., in the MPT and computer‐based learning environment) – interest did not reliably predict test achievement. This suggests that triggered interest might serve as a strong predictor of learning success particularly when individual interest is high (Jansen et al., 2016; Linnenbrink‐Garcia et al., 2013).

As learning progresses, effort facilitates the mobilization of resources, becoming increasingly important for learning success. According to Hypothesis 2, actual effort emerged as a positive and significant predictor of learning success across all three tasks. Furthermore, in accordance with Hypothesis 4, it was found that the influence of actual effort on learning success in CPT and the computer‐based learning environment was significantly stronger than that of actual interest.

Our findings suggest that interest alone might not be sufficient to successfully master learning tasks. We found that effort plays a crucial role in the learning process in mobilizing additional resources to complete the task. Those who exerted more effort and utilized their resources more effectively achieved better learning outcomes. These findings align with motivational intensity theory (Brehm & Self, 1989), suggesting that effort leads to better results when perceived as meaningful and justified. Therefore, effort can be predictive of learning success, especially during the learning process, when learners actively make an effort. As they gain the experience that it is worth investing more effort in a learning task, actual interest becomes less important. Effort is a consistently positive predictor of learning success, which aligns with previous empirical findings (Jin, 2024; Silm et al., 2020; Zhang et al., 2023).

In the learning process, triggered interest – which is influenced by individuals' previous experience with similar tasks – is initially important, as suggested by our results. Effort is then exerted based on triggered interest. Dewey (1913, p. 59) already argued that “the effort needed is secured when the activity in question is of such positive and abiding interest as to arouse the person to clearer recognition of purpose and to a more thoughtful consideration of means of accomplishment.” Triggered interest might therefore encourage reflection on the purpose and the means of achieving the goal, which in turn facilitates actual effort in the learning process. Moreover, according to the mood–behaviour model (Gendolla, 2000, 2025), a positive mood has a favourable effect on the expenditure of effort if task difficulty is high. Since interest generates a positive mood, it can positively influence effort. Additionally, this aligns with motivational intensity theory (Brehm & Self, 1989) if interest is understood as a form of potential motivation. When potential motivation and thus interest is high, individuals are willing to invest more effort in a task. This is further supported by other findings suggesting that interest not only positively influences learning but also contributes to increased effort (Jansen et al., 2016; Marsh et al., 2005). Additional evidence comes from our analysis of indirect effects: The indirect effect of triggered interest on test achievement through actual interest is not significant (Study 1: CPT and Study 2). This suggests that triggered interest does not simply carry forward over time or automatically translate into stronger actual interest. Rather, the effects of actual interest are mediated through actual effort. Notably, the indirect effect of actual interest on test achievement through actual effort is significant across all three learning tasks. Thus, actual interest may not be less predictive than actual effort but may function as a more distal predictor.

The interplay of interest and effort is therefore evident in all three learning tasks, although these vary greatly and differ considerably in terms of the learners' previous experience. Our findings are consistent with Dewey's (1913) understanding of interest and effort as a shared growth activity. Interest and effort collectively ensure that learners engage with a new learning environment and invest effort in navigating the learning process.

### Limitations and directions for future research

Our studies have some limitations that should be addressed in future research projects. Modified learning tasks, a more rigorous control of third variables, and alternative measurement methods could contribute to a better generalisability of the results. We would like to briefly outline these suggestions in more detail.

First, learning tasks could be developed that provide even stronger motivational stimulation. These could be tasks with greater relevance to everyday life, better reflecting students' typical learning experiences. These intrinsically motivating learning activities could be associated with higher and more sustained interest, which, in turn, would also alter the feeling of effort.

Second, learning tasks spanning a longer time frame should be tested. Even within short time spans, an interplay of interest and effort in the learning process has been demonstrated. However, it would be informative to examine how the relationships between interest, effort, and test achievement change over the course of a longer learning process. Especially since reverse paths are also conceivable – that is, actual effort may lead to increased actual interest – it should be noted that the present study did not test this pathway. Future research could explore this possibility to further clarify the directionality of the relationship between interest and effort.

Third, in addition to prior knowledge, other control variables should be considered to ensure that the relationships identified are stable. Since current motivation arises from an interaction between stimuli of the learning situation and individual personality traits (Urhahne & Wijnia, 2023), future studies could incorporate additional situational factors or personality traits, such as the academic self‐concept, the Big Five, or achievement goal orientations. As this study used a modelling approach, causal conclusions cannot be drawn. Future research could further control for these variables within a controlled experimental design to examine causal mechanisms (Mayer, 2023).

Fourth, variation in the measurement methods could provide further insights. In both studies, actual interest and actual effort were subjectively measured using semantic differentials to capture the constructs as time‐efficiently as possible during learning. An objective measure for effort intensity could provide further insights (Gendolla et al., 2012). An alternative approach for future research could be the experience sampling method, which would enable a more precise mapping of changes in motivation from moment to moment (Järvinen et al., 2022).

## CONCLUSIONS

By incorporating effort, the current theory of interest has been extended and enriched (Renninger & Hidi, 2022; Thoman et al., 2017). Both motivational constructs are interrelated and, together, can positively influence learning processes and learning outcomes (Dewey, 1913; Milyavskaya et al., 2021). It has been shown that initially, the stimulation of interest plays an important role in engaging with the learning task and working on it attentively and with concentration. However, as learning progresses, effort becomes increasingly important and can supplement interest‐based learning when the further mobilization of mental resources is required to master the learning task. These assumptions were confirmed by the findings of both studies.

The results clearly indicate that interest and effort should be viewed as two components of a common growth activity (Dewey, 1913). Effort is a crucial element in the organization of successful learning processes and should not be overlooked. Consistently high performance throughout the entire learning process cannot be sustained by interest alone. While situational interest can be stimulated through novel stimuli, academic effort must also be cultivated by making learners aware of their learning goals and guiding them along purposeful learning pathways (Dewey, 1913). In this way, the interplay between interest and effort can fully realize its potential.

## AUTHOR CONTRIBUTIONS

**Laura Kehle:** Conceptualization; writing – original draft; methodology; investigation; validation; visualization; software; formal analysis; project administration; data curation; resources. **Detlef Urhahne:** Supervision; writing – review and editing.

## CONFLICT OF INTEREST STATEMENT

We have no known conflict of interest to disclose.

## Data Availability

The data will be available upon request from the authors.
